# Virtual Double-System Single-Box: A Nonequilibrium Alchemical Technique for Absolute Binding Free Energy Calculations: Application to Ligands of the SARS-CoV-2 Main Protease

**DOI:** 10.1021/acs.jctc.0c00634

**Published:** 2020-10-22

**Authors:** Marina Macchiagodena, Marco Pagliai, Maurice Karrenbrock, Guido Guarnieri, Francesco Iannone, Piero Procacci

**Affiliations:** † Dipartimento di Chimica “Ugo Schiff”, Università degli Studi di Firenze, Via della Lastruccia 3, 50019 Sesto Fiorentino, Italy; ‡ ENEA, Portici Research Centre, DTE-ICT-HPC P.le E. Fermi, 1, I-80055 Portici (NA), Italy

## Abstract

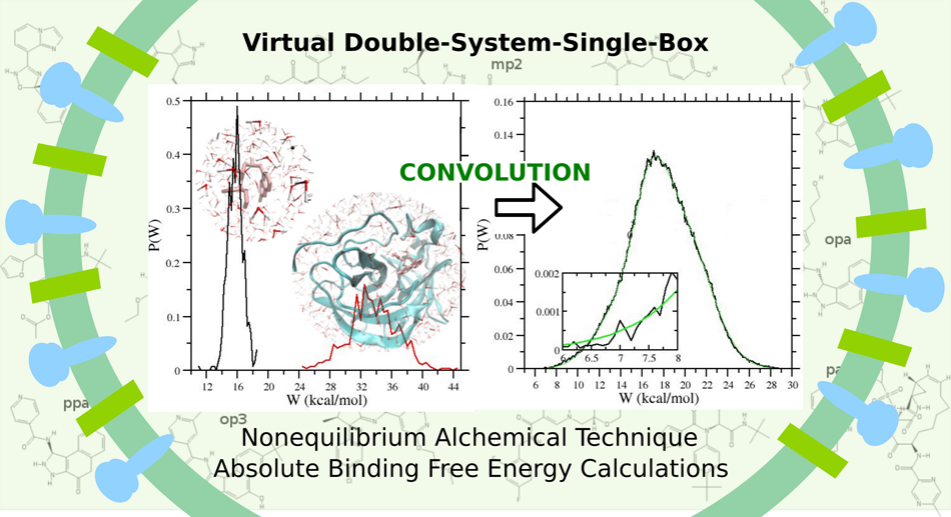

In the context of drug–receptor binding affinity calculations using molecular dynamics techniques, we implemented a combination of Hamiltonian replica exchange (HREM) and a novel nonequilibrium alchemical methodology, called virtual double-system single-box, with increased accuracy, precision, and efficiency with respect to the standard nonequilibrium approaches. The method has been applied for the determination of absolute binding free energies of 16 newly designed noncovalent ligands of the main protease (3CL^pro^) of SARS-CoV-2. The core structures of 3CL^pro^ ligands were previously identified using a multimodal structure-based ligand design in combination with docking techniques. The calculated binding free energies for four additional ligands with known activity (either for SARS-CoV or SARS-CoV-2 main protease) are also reported. The nature of binding in the 3CL^pro^ active site and the involved residues besides the CYS–HYS catalytic dyad have been thoroughly characterized by enhanced sampling simulations of the bound state. We have identified several noncongeneric compounds with predicted low micromolar activity for 3CL^pro^ inhibition, which may constitute possible lead compounds for the development of antiviral agents in Covid-19 treatment.

## 1 Introduction

As the whole world is currently plagued by the Covid-19 pandemic, the race to identify an effective antiviral agent for SARS-CoV-2 is frantically ongoing. Among the viral functional proteins, the main protease 3CL^pro^^1,2^ constitutes a very attractive biomolecular target for drug design. Indeed, inhibition of 3CL^pro^ using small molecules is currently the main goal of the crowdsourcing drug discovery initiative for pandemics.^3^ This protein is responsible for the cleavage of pp1a and pp1ab large polyproteins expressed by the virus RNA upon cell entry. The cleavage on multiple points of these polyproteins releases in the infected cell mature nonstructural proteins that are important for virus replication. For example, the virus replication machinery itself, the RNA-dependent RNA polymerase (RdRp), is generated upon 3CL^pro^ cleavage of pp1a, pp1ab.^4,5^ It is hence expected that a potent and specific inhibitor of 3CL^pro^ can effectively block the viral replication once the virions have entered the cell.

One of the main approaches for anti-Covid-19 drug development is based on past experience on the SARS 2003 outbreak. Indeed, the RNA of SARS-CoV-2 and of SARS-CoV share nearly 80% genomic sequence identity.^6^ The main protease of SARS-CoV^7^and SARS-CoV-2^2,5,8^ differs by only 12 residues, with none of these differing residues being directly involved in the catalytic site.^9^ It is hence expected that inhibitors for SARS-CoV 3CL^pro^ bind effectively the strictly related SARS-CoV-2 main protease. 3CL^pro^-based drug discovery for SARS^10^ was mainly directed toward the so-called covalent Michael inhibitors^11,12^ via electrophilic attack on the cysteinate of the 3CL^pro^ catalytic CYS145–HIS41 dyad. New irreversible covalent inhibitors for SARS-CoV-2 3CL^pro^ were recently proposed in ref (5). On the other hand, the consensus in drug discovery leads to excluding electrophiles from drug candidates for reasons relating to safety and adverse effects such as allergies, tissue destruction, or carcinogenesis.^13^ Noncovalent inhibitors for SARS-3CL^pro^ were first identified in ref (14) and later characterized in ref (15), leading to the synthesis of ML188 with a measured inhibitory activity of 2 μM.

The second arm of the current research for drug therapy of Covid-19 is focused on drug repurposing, hence testing approved compounds with little (and known) side effects and with a minimal development cost for off-label use. In this context, a very extensive and complete multicentric study of the SARS-CoV-2 protein interaction map^16^ revealed human targets for drug repurposing. The targets were in this case human proteins (such as σ-receptors or bromodomains) characterized as playing an important part in the viral interactome.

From a computational standpoint, docking studies on 3CL^pro^ started to appear immediately after the release in mid-February of the PDB structure of 3CL^pro^.^8^ Docking is one of the main computational tools used in compound triaging in the cited COVID-19 moonshot worldwide initiative.^3,17^ As of today, more than 20 entries on 3CL^pro^ ligand screening using docking either alone or in combination with structure-based or data-driven approaches have been published so far, according to the Scopus database. Many more, deposited in preprint servers, are awaiting for the peer-review process to complete. We were among the first to deposit on the arXiv server a study^9^ on 3CL^pro^ inhibitors using a multimodal structure-based design^18^ in combination with molecular docking.^19^ Evaluation of docking scores is fast and docking is indeed an invaluable tool for a plausible pose prediction and for a semiquantitative assessment of the inhibitory power of a ligand. However, binding free energies solely based on docking are, in general, considered not sufficiently reliable as this technique exhibits by design a major weakness, that is, the partial neglect of the ligand and receptor Boltzmann-weighted conformational disorder as well as of solvent-related microsolvation phenomena, eliciting the crucial and elusive entropy contribution to the binding free energy.^20^

Pose prediction using Docking can be assessed and refined using molecular dynamics (MD) advanced techniques with full atomistic detail such as free energy perturbation^21^ (FEP) or thermodynamic integration (TI).^22^ Surprisingly, to our knowledge, not many FEP studies combined with alchemical transformation^23,24^ on 3CL^pro^ binders appeared so far in the literature.^25^ Possibly, this is due to the fact that FEP-based modern alchemical techniques^26^ are costly and generally applied to relative binding free energies on strictly congeneric series, with hence a limited value in drug discovery campaigns based on *de novo* design. On the other hand, FEP calculations for absolute binding free energies (ABFEs) are still quite rare as they face serious sampling problems due to the mobility of the ligand in the binding site, in general, especially for low-coupling alchemical states.^27^

In the past years, we have been developing a nonequilibrium (NE) variant^28−32^ of alchemical transformations, whereby the ligand is rapidly decoupled from the environment in a swarm of rapid independent trajectories producing a NE work (NEW) distribution histogram, related to the decoupling free energy via well-known NE theorems. These NE alchemical decoupling trajectories, typically lasting less than 1 ns, start from equilibrium phase space points that are sampled using very efficient and highly parallelizable enhanced sampling techniques, such as Hamiltonian replica exchange with torsional tempering.^33^ The NEW approach allows us to release altogether the artificial conformational and orientational restraints in the bound and unbound states that are commonly used in FEP calculation to facilitate sampling^34^ at the price of focusing on a pose that can be suboptimal.^35^ The NEW approach turned out to be among the top-performing techniques assessed in recent SAMPL challenges^36,37^ for blind absolute binding free energy predictions. Here, we have implemented yet a new improved variant of the NEW approach, called virtual double-system single-box (vDSSB) on the basis of the recent remark on the so-called DSSB approach used in the latest SAMPL challenge.^38^

NEW-vDSSB has been applied to the calculation of the dissociation free energy for a total of 21 3CL^pro^ noncovalent complexes, with some of the ligands identified in ref (9) and with some of their analogues, as well as for few ligands with recently measured activity for 3CL^pro^.^17^ The common binding pattern of these ligands in the shallow and wide binding pocket of 3CL^pro^ is analyzed in detail using enhanced sampled configurations, providing valuable information on drug design against the 3CL^pro^ target. A few compounds with predicted submicromolar activity have been designed, hopefully providing promising leads for an effective medicinal chemistry campaign for the identification of a therapeutic agent for Covid-19.

The paper is organized as follows: we first lay out with some detail the theoretical background for the NEW-vDSSB determination of the drug–receptor dissociation free energy. We then describe the 3CL^pro^ system and its function, giving a rationale for its modelization in a drug design study. Technical details for MD, in general, as well as for enhanced sampling and nonequilibrium simulation approaches are described in Section 4. Computed dissociation free energies for the 21 scrutinized complexes using NEW-vDSSB along with a detailed analysis of the binding pattern is reported in Section 5. Section 6 presents concluding remarks.

## 2 Theoretical Background

The NEW method has been developed in the past two decades in the context of binding free energy calculations for drug–receptor systems as a nonequilibrium variant of the free energy perturbation^21^ method with stratification.^23^ In the nonequilibrium double-system single-box approach^39^ (NEW-DSSB), a bound ligand is annihilated in the receptor binding site, while a second unbound ligand, kept using restraints far away from the protein, is simultaneously grown in the bulk solvent in a series *n* of independent alchemical trajectories, where the alchemical parameter λ regulating the ligand–environment coupling level^23,24^ is varied in the range [0,1] (or [1,0]) according to a common time schedule. The alchemical DSSB approach was recently applied^40^ also in the context of the equilibrium alchemical FEP with λ-stratification.^21,41,42^ Provided that the two ligands do not feel each other and the unbound ligand is constantly surrounded by a sufficiently thick layer of the solvent, the computed work distribution in NEW-DSSB is directly related to the dissociation free energy of the ligand.^43,44^ NEW-DSSB works well if the simulation box is large enough so that the growth and annihilation work of the two distant ligands can be assumed to be uncorrelated. Alternatively, in the single-box double-system approach (SSDB), one can compute, in two different alchemical processes, the decoupling and recoupling free energy for the bound ligand and for the ligand in bulk, obtaining the binding free energy as the sum of these two contributions.

In NEW-based alchemical techniques, the dissociation free energy (except for a volume correction^43,44^) can be recovered from the NE work distributions (obtained computing the work for each of the *n* independent alchemical trajectories) as
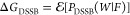
1
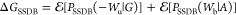
2where *P*(*W*|*F*), *P*(−*W*_u_|*G*), and *P*(*W*_b_|*A*) are the work distributions for the double system and for the unbound (growth) and bound (annihilation) states of the single system, respectively, and  is a functional of the work distribution of the NE process, yielding the estimate of the free energy difference, Δ*G*, of the process. For unidirectional processes,  corresponds to the Jarzynski estimate^45^
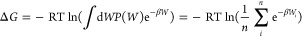
3or to the Gaussian estimate^46^
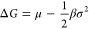
4eq. 4 is valid when *P*(*W*) is the normal distribution, *n*(*W*,μ,σ) with mean and variance μ and σ^2^. *W*_diss_ = μ – Δ*G* corresponds to the dissipated work in the NE process, which in the case of the normal distribution is given by *W*_diss_ = (1/2)βσ^2^. It should be noted that, for normal distributions, the Crooks theorem^47^ implies that the forward distribution (*P*_F_(*W*)) and reverse distribution (*P*_R_(−*W*) done with inverted time schedule) are mirror-symmetric with respect to their unique crossing point, *W*_c_ = Δ*G*, and that their maxima are βσ^2^ = 2*W*_diss_ far apart from each other.^46^ The inverse of the dissipated work in Gaussian (Markovian) alchemical processes is a linear function^48^ of the duration τ of the NE alchemical processes. Both accuracy and precision decline with increasing dissipation^48,49^ (or, equivalently, faster NE processes). Note that we have assumed a forward process in NEW-DSSB that corresponds to the annihilation of the bound ligand and growth of the ligand in the bulk. The associated work for binding ligands is positive, corresponding to positive dissociation free energies. This choice is dictated by the fact^27^ that, in the reverse process, the starting states of the decoupled and weakly restrained ligand in the binding pocket are characterized by a high conformational disorder, with most of the generated NE recoupling trajectories producing suboptimal poses in highly dissipative processes. This is a typical situation in NE transformations involving the entrance into a free energy funnel.^50^

For non-Gaussian bidirectional processes done with time-inverted protocols, eqs 1 and 2 take the form of
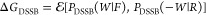
5
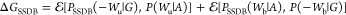
6where *F* and *G* denote the forward and reverse processes and the functional  is the Bennett acceptance ratio (BAR) estimate.^51,52^ Note that in eq 2, referring to the unidirectional SSDB estimates, if the ligand bears a net charge, one must add an analytic correction to Δ*G* due to the annihilation of the net charge in the two independent alchemical processes when using particle mesh Ewald (PME)^53^ with a neutralizing background plasma.^54,55^ The correction exactly cancels out in the unidirectional DSSB processes and in bidirectional SSDB. At constant τ, the BAR bidirectional estimate is more accurate than unidirectional estimates, provided that there is sufficient overlap between the forward and reverse work distributions.^48,49,56^

As discussed in ref (57), for the case of the NEW applied to Gaussian processes, the DSSB/SSDB efficiency ratio *R* can be shown to be given by
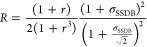
7where *r* = *L*_s_/*L*_b_(*r* ∈ (0, 1]) is the ratio between the side lengths for the optimal box of the bound and unbound systems. According to eq 7, DSSB is more efficient than SSDB (*R* < 1) when the variance of the NE work distributions (assumed to be equal for the growth and annihilation processes) are small and *r* ≃ 1. The eciency gain in DSSB becomes insignicant when the optimal box for the bound system is signicantly larger than that of the un- bound ligand (which occurs systematically in drugreceptor systems) and/or in the case of highly dissipative processes.

However, since in SSDB the work values in the two alchemical processes for bound and unbound states are independent random variables (RV), one can emulate the DSSB by combining each value of the RV *W*_b_(*A*) with each value of the RV *W*_u_(*G*), hence obtaining *n*^2^ work RV’s *W* = *W*_b_(*A*) + *W*_u_(*G*) instead of the original *n*. This process corresponds to evaluating the convolution *P*(*W*|*F*) = (*P*_b_*P_u_) (*W*|*F*) = ∫d*wP*_b_(*W*|*A*) *P*_u_(*W* – *w*|*G*), thus leading to the following equations
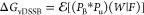
8
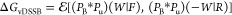
9where eqs 8 and 9 refer to the unidirectional and bidirectional estimates, respectively, and (*P*_b_*P_u_)(−*W*|*R*) = ∫d*wP*_b_(−*W*|*G*)*P*_u_(−*W* + *w*|*A*). When using a bidirectional approach in vDSSB, due care must be taken in using a time-inverted protocol for both legs (bound and unbound) of the alchemical process.

The advantages of eq 8 over eq 1 are evident. First, the convolution of the *P*_u_ and *P*_b_ distribution boosts the statistics, increasing significantly the resolution of the vDSSB work distributions, *P*(*W*|*F*) and *P*(−*W*|*R*). The convolution (*P*_b_*P_u_) (*W*|*F*) can now be computed using a sample of *n*^2^ work outcomes at the cost of *n* bound and unbound trajectories. In the second instance, at variance with DSSB, where a common time protocol for the process is adopted, in vDSSB, the time protocol for the bound and unbound states alchemical simulations can be chosen independently with no violation of the Crooks or Jarzynski theorems. In particular, the alchemical process for the unbound state can be done using a much faster rate with respect to that of the bound state since the dissipation in the anisotropic environment of the binding pocket is, in general, much higher than that experienced by the ligand in the isotropic environment of the bulk solvent. Third, in vDSSB, the optimal box size can be chosen according to the physical dimension of the solute. For the ligand in bulk, the box can be chosen much smaller than that of the ligand in the bound state, with a significant gain in the computational efficiency (up to 35% according to eq 7).

## 3 3CL^pro^–Ligand Complexes

### 3.1 CL^pro^ Structure and Function

**Figure 1:**
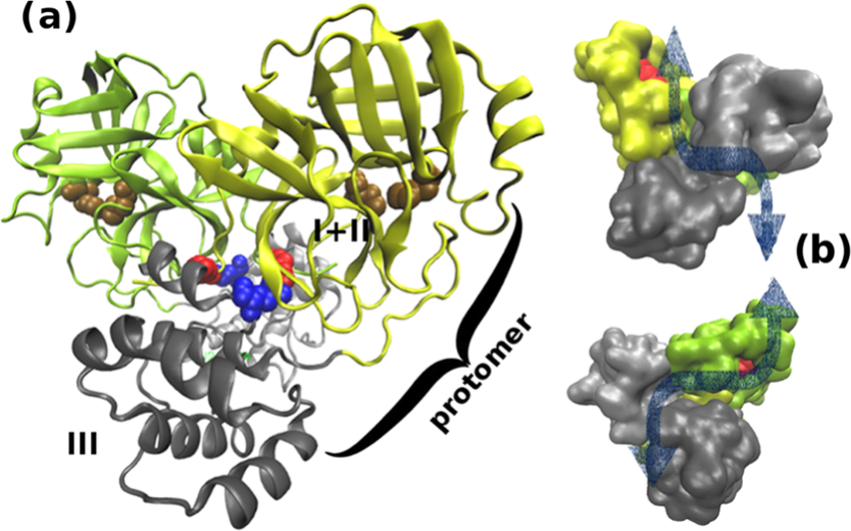
(a) Three-dimensional (3D) structure of the SARS-3CL^pro^ dimer.^5^ Domains I + II and III are in yellow and gray, respectively. The catalytic sites (VdW representation) are in ocher. The salt bridges GLU290-ARG4, connecting domains III of the protomers, are in blue (GLU290) and red (ARG4), respectively. (b) Surface representation of upper and lower sides of the dimer highlighting the clefts for pp1a/pp1ab adhesion. The catalytic pocket is shown in red.

3CL^pro^ acts as a dimer.^58^ The monomer is in turn composed of two loosely coupled units, the chymotrypsin-like domains I + II (residues 1–197), harboring the catalytic site, and the cluster of helices domain III (residues 198–304), regulating dimerization via two intertwined salt bridges involving ARG4(A)-GLU290(B) and GLU290(A)-ARG4(B) of the A and B protomers, as shown in Figure 1a. The dimer is characterized by two symmetric extended clefts (shown as blue arrows in Figure 1b) for pp1a, pp1ab adhesion. Each dimer cleft ends at the solvent-exposed catalytic site with the CYS145–HIS41 proteolytic dyad. The two catalytic dyads, on the opposite sides of the dimer, very likely act independently for maximizing the catalytic efficiency. Polyproteins are cleaved by betacoronavirus SARS-CoV and SARS-CoV-2 main proteases at the glutamine level in the general sequence X(L/F/M)Q↓(G/A/S)X, where X is any residue.^4^ The cleavage sites in the SARS-CoV-2 pp1ab sequence^59^ are reported in Table 1.

**Table 1:** SARS-CoV-2 pp1ab Cleavage Sites

res(Q)	seq	gap
3263	LQS	3263
3569	FQS	306
3922	MQG	353
3942	LQA	20
4253	LQA	311
4392	LQS	139
5324	LQA	932
5605	LQG	281
5925	LQA	320
6179	LQS	254
6452	LQS	273
6798	LQS	346

It is worth noting the hydrophobic character of most of the Q-neighboring amino acids, indicating that docking of the polyprotein at the proteolytic site is likely to occur via complementary hydrophobic interactions. In the light of the dimer peculiar structure and functional mechanism, with the solvent-exposed and distal proteolytic sites, the dissociation constants for 3CL^pro^ ligand association can be effectively and reliably computed by modeling only the domain I + II of one protomer for the bound state (residues 1–197). We must nonetheless stress that the computed Δ*G* pertains to the associations of the ligand with one protein, irrespective of the state of association of the protein. At free ligand concentration equal to *K*_d_ ≡ e^–Δ*G*/*RT*^, i.e., when half of the protein molecules are inhibited, the probability to have both monomers inhibited on a catalytically active dimer is equal to 1/4, whatever the dissociation constant of the dimer is,^58^ hence the need for identifying nanomolar or sub-nanomolar inhibitors of 3CL^pro^.

### 3.2 3CL^pro^-Tested Compounds

**Figure 2:**
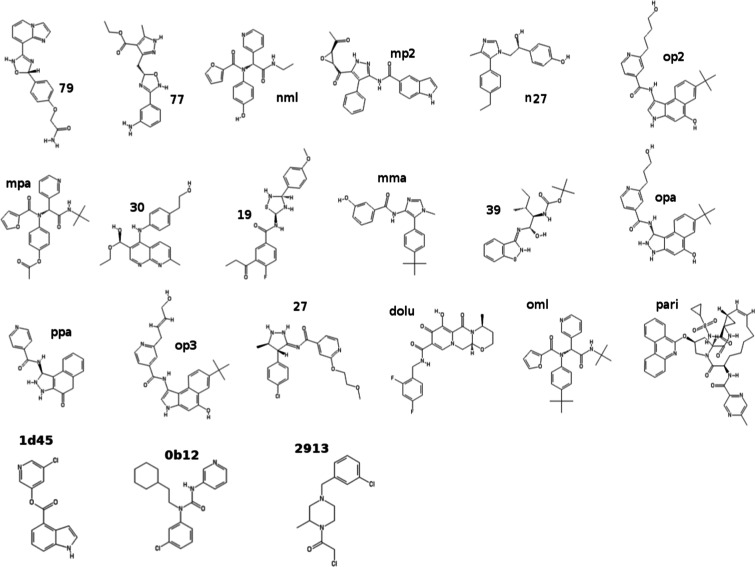
Ligands for 3CL^pro^.

In the present study, we have computed, using NEW-vDSSB, the absolute binding free energies (ABFEs) of the 21 compounds reported in Figure 2. Compounds **79**, **27**, **19**, **77**, and **39** were previously identified^9^ as probable binders (8 < Δ*G* < 9 kcal/mol) using a multimodal structure-based design^18^ in combination with molecular docking.^19^ Compound **nml** (ML188) is a known inhibitor^15^ for SARS-CoV 3CL^pro^ with micromolar activity. Compounds **dolu** and **pari** are Dolutegravir and Paritaprevir and are recently identified^60^ using virtual screening (Docking and Standard MD) as candidate lead compounds for SARS-CoV-2 3CL^pro^ and 2′-OMTase inhibition. All other compounds have been designed in this study by analyzing the binding pattern from bound-state enhanced sampling trajectories (vide infra). Among the compounds reported in Figure 2, only **dolu**, **pari**, and **oml** are commercially available as reported by the ZINC database.^61^ The activity (IC50) of the compounds **1d45**, **0b12**, and **2913** for the inhibition of the SARS-CoV-2 main protease was recently measured in the context of the Covid-19 Moonshot initiative.^17^

## 4 Methods

### 4.1 Overview

We started by docking the OpenBabel-generated^62^ 3D structure of the ligands to the PDB structures of 3CL^pro^ (domains I + II only) and PL^pro^ using Autodock4.^19^ The optimal initial docking pose was found by running 50 minimization rounds with the center of mass (COM) of the fully flexible ligand placed within a 15 Å side-length cubic box centered at the protein active sites. The latter were identified by the midpoint vectors connecting the α carbons of the CYS145–HIS41 catalytic dyad in 3CL^pro^. The 3CL^pro^ target is rigid to avoid sampling of unlikely nonopen conformations of the active site region.

The so-generated initial structures of the complexes were first equilibrated in a cubic box of appropriate size, filled with TIP3P^63^ explicit water molecules, by running short simulation (100 ps) in the NPT ensemble. The resulting solvated complexes were then fed to the ORAC MD program^64^ for the Hamiltonian replica exchange (HREM) sampling of the bound states using a powerful torsional tempering scheme in the binding site region engaging only eight replicas.^33,65^ For each complex, we collected 540 configurations sampled at regular intervals during the 25 ns NPT simulation of the HREM target (unscaled) state (*T* = 300 K and *P* = 1 atm). From these HREM-harvested equilibrium configurations, we launched, on a single parallel job, a swarm of 540 independent alchemical nonequilibrium (NE) trajectories^31,32^ where the ligand–environment interactions were rapidly decoupled in 0.36 ns, eventually producing a ligand annihilation work distribution. During the HREM and NE simulations, the ligand was prevented to drift away from the active site using a weak harmonic restraint between the centers of mass (COM) of the ligand and the receptors.^31^ No orientational or conformational restraints are imposed on the ligand, which is free to explore all of the poses and orientations within the allowance volume^43,66^*V* = (2π*RT*/*K*)^3/2^ set by the weak COM–COM restraint.

The recoupling work distribution of the ligand in bulk solvent was obtained using fast-growth (0.36 ns) alchemical simulations. The starting configurations, in this case, were generated by combining 540 solvent-decoupled conformations of the ligand, sampled in an 8 ns HREM simulation with torsional tempering of the isolated (gas-phase) molecule, with equilibrated structures of pure TIP3P water molecules in standard conditions.

The standard dissociation free energies, Δ*G*_0_, were computed using the Jarzynski estimate^45^ equation (eq 8), evaluated on the work distribution obtained by combining the negative growth work values of the ligand in bulk with the positive decoupling work values of the ligand in the bound state, and by adding a standard state binding site volume correction.^31^ The 95% confidence interval of the predicted dissociation free energies was obtained by bootstrapping with resampling on the two independent sets of growth and decoupling work values, before convoluting the data. All MD calculations were performed using the program ORAC^64^ on the CRESCO6 high-performance computing (HPC) facility located in Portici (Italy) and managed by ENEA.^67^ Details of the MD settings, HREM parametrization, and NE protocols are reported subsequently, in Sections 4.2–4.4.

### 4.2 MD: General Settings

All simulations for the bound and unbound states were done in the NPT isothermal–isobaric ensemble under periodic boundary conditions on cubic or orthogonal MD boxes with explicit TIP3P water molecules. We used the AMBER99SB-ILDN force field^68^ for 3CL^pro^. Default protonation states (pH = 7.6) of titratable residues were used. The ligands were described using the GAFF2 force field, with atom types and AM1/BCC charges assigned using the PrimaDORAC web interface.^69^ The potential parameters for all ligands of Figure 2 are provided in the Supporting Information (SI). The external pressure was set to 1 atm using a Parrinello–Rahman Lagrangian^70^ with isotropic stress tensor. The temperature was held constant at 300 K using three Nosé–Hoover thermostats coupled to the translational degrees of freedom of the systems and the rotational/internal motions of the solute and the solvent. Constraints were imposed only to X–H bonds, with X being a heavy atom. The equations of motion were integrated using a multiple time step r-RESPA scheme^71^ with a potential subdivision specifically tuned for biomolecular systems in the NPT ensemble.^70,72^ The long-range cutoff for Lennard–Jones interactions was set to 13 Å. Long-range electrostatics were treated using the smooth particle mesh Ewald method,^53^ with an α parameter of 0.38 Å^–1^, a grid spacing in the direct lattice of about 1 Å, and a fourth-order B-spline interpolation for the gridded charge array. The net charge on the system (due to proteins) was neutralized by a uniform neutralizing background plasma as it is customary when using PME.^54^

### 4.3 HREM Simulations

The HREM simulations of the bound state were run by launching, in a single parallel job, 12 batteries of independent Hamiltonian replica exchange simulation with eight replicas, for a total of 96 MPI instances and a total simulation time on a per complex basis of ≃0.2 μs (25 ns on the target state). In each eight-replica battery, we used a torsional tempering scheme (including 14 nonbonded interactions) with a maximum scaling factor *s* = 0.2 corresponding to a torsional temperature of 1500 K. The “hot” region includes all residues with at least one atom at a distance of less than 4.2 Å from any atom of the ligand, as found in the best docking pose. The scaling factors, *s_m_*, along the eight replica progression are computed according to the protocol *s_m_* = *s*^(*m*–1)/7^. The exchange was attempted every 15 fs (every large time step^73^), and the average exchange rate was, in all cases, around 15–20% with round-trip times of around 0.3–0.4 ns.

The ligand was weakly tethered in the binding site via a harmonic restraint potential between the COM of the ligand and that of the protein, with equilibrium distance corresponding to the COM–COM distance of the lowest energy docked pose and a force constant of 0.05 kcal mol^–1^ Å^–2^.

For setting up the starting configurations of the decoupled ligand in bulk, we first harvested 540 configurations of the isolated (gas-phase) molecule via an 8 ns (target state) HREM simulation using four replicas with torsional tempering with a minimum scaling factor of *s* = 0.1, corresponding to a torsional temperature of 3000 K, and using the protocol *s_m_* = *s*^(*m*–1)/3^, *m* = 1...4 along the four replica progression. The 540 sampled gas-phase ligand conformations, with random orientations and positions, were combined with a single equilibrated sample of about 1800 water molecules in standard conditions in a cubic box, producing 540 starting configurations of the decoupled (ghost) ligand in the bulk.

### 4.4 NE Alchemical Simulations

For the ligand in the bound state, the alchemical annihilation simulations were performed starting from the λ = 1 (fully coupled) equilibrium configurations collected in the preceding HREM step. NE annihilation trajectories were run for 360 ps: in the first 120 ps, the electrostatic interactions were linearly switched off; in the following 120 ps, two-thirds of the Lennard–Jones potential was turned off, and in the last 120 ps, the one-third residual was finally switched off.

A time-inverted protocol was adopted for the ligand in the bulk state (u state); in this case, the fast-growth alchemical simulations were started from the λ = 0 (fully decoupled) and the NE trajectories were run for 360 ps. In the first 120 ps, one-third of the Lennard–Jones potential was turned on. In the following 120 ps, the Lennard–Jones potential was switched on completely. In the last 120 ps, the electrostatic interactions were linearly turned on. All of the simulations for computing inhibitor constants are done using the program ORAC.^64^ The program is distributed under the GPL and can be downloaded free from the website www.chim.unifi.it.

## 5 Results and Discussion

### 5.1 Binding Free Energy Results

**Figure 3:**
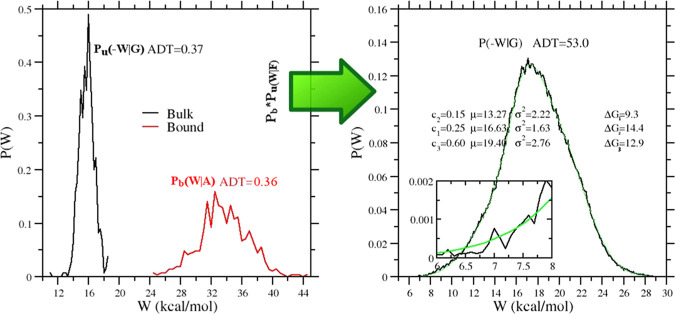
Left panel: growth, *P*_u_(−*W*|*G*), and annihilation, *P*_b_(−*W*|*G*), work distributions computed using 540 work values for the **mma** ligand (see Figure 2). Right panel: convolution work distribution (*P*_b_*P_u_)(*W*|*F*) for the forward process (black) and expectation-maximization (EM) fit (green) with three components, with Δ*G*_*i*_ = μ_1_ – βσ_*i*_^2^/2. The inset shows a highlighted view of the left tail of the distribution and the EM fit.

In Figure 3, we illustrate the vDSSB approach for evaluating *P*(*W*|*F*), referring to the **mma** ligand. The annihilation (bound ligand) and growth (bulk ligand) work distributions are constructed by computing the work in a few hundreds of NE alchemical decoupling and recoupling 0.36 ns trajectories, respectively. Note that the dissipation for the growth process in bulk is much smaller than that for the annihilation in the bound state at equal time τ of the NE processes, leading to a systematic disparity in the histogram resolution. The mean-variance, σ^2^, values for the growth and annihilation distributions *P*_u_(−*W*|*G*) and *P*_b_(*W*|*A*) are 1.1 and 15.3 kcal^2^ mol^–2^, respectively. In the reported typical example for the **mma** ligand, both distributions “look” Gaussian and they both amply satisfy the Anderson Darling (AD) test for normality.^74^ We recall that the AD test gives only the probability for rejecting the null hypothesis (i.e., the work values are normally distributed) but does not provide, like any other normality test, any certitude on the correctness of the null hypothesis.

As a matter of fact, the convolution of the two distribution (right panel in Figure 3), by boosting the statistics and the resolution of the work histogram, visually reveals the non-Gaussian character of the resulting *P*(*W*|*F*), which, in the case of **mma**, exhibits a marked negative skewness. In this event, eq 4 cannot be used. The high number (*n*^2^) of work data for the construction of *P*(*W*|*F*), with good sampling also in the left tail of the distribution, allows for a reliable estimate of the free energy based on the Jarzynski exponential average,^75^eq 3. Alternatively, the convolution distribution can be decomposed into *c* normal components, *P*(*W*|*F*) = ∑_*i*_^*c*^*w*_*i*_*n*(*W*,μ_*i*_,σ_*i*_), using the expectation-maximization (EM) algorithm,^76,77^ with ∑_*i*_^*c*^*w*_*i*_ = 1. By the Crooks theorem, it can be shown^50^ that the free energy functional for Gaussian mixtures is given by
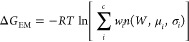
10The EM algorithm is very efficient in fitting the convolution histogram, as Figure 3 shows. For the assessment of the confidence level for the estimate (whether Jarzynski or EM) based on the combination of the work data, some important remarks are in order. Bootstrapping the *n*^2^ sample of the combination yields an unrealistically small error. As errors in the coarse-grained distributions *P*_u_(−*W*|*G*), *P*_b_(*W*|*A*) can be propagated in the high-resolution convolution, the uncertainty of the estimate must be evaluated by bootstrapping independently the growth and annihilation work samples and then combining the data using either the Jarzynski or EM functional of the bootstrapped convolution.

**Figure 4:**
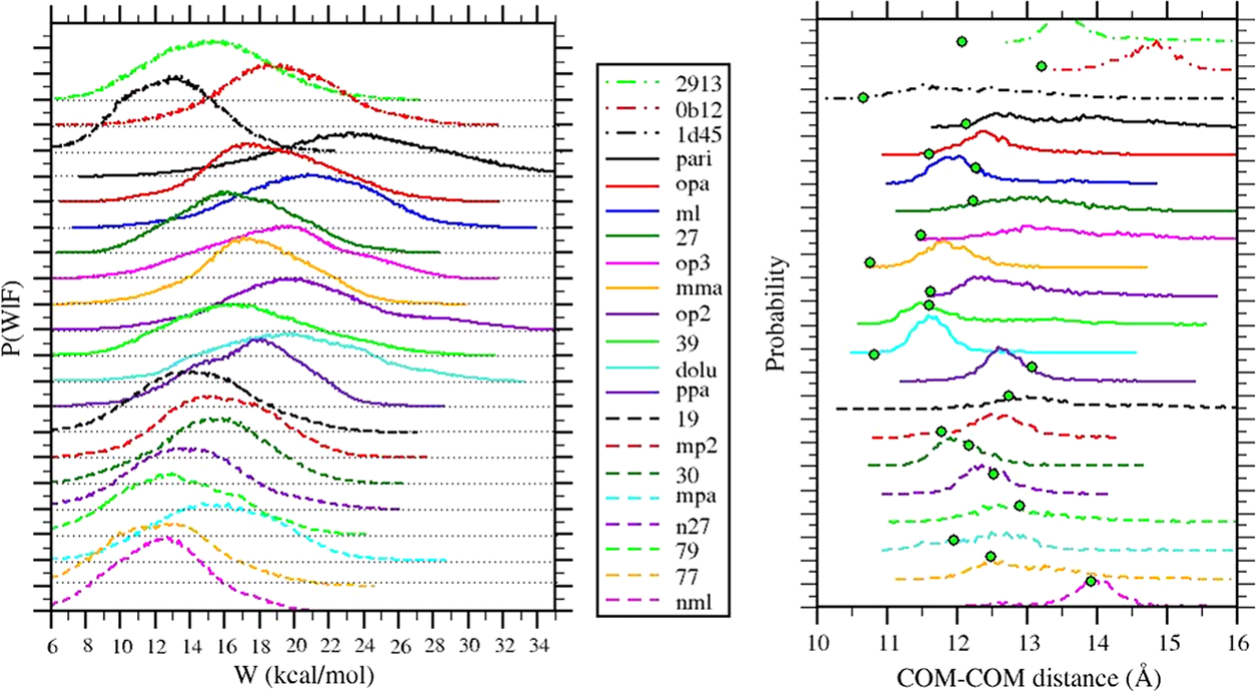
Left panel: work distributions for the vDSSB approach for the ligands reported in Figure 2 as obtained from the NE simulations in the bulk (growth) and in the 3CL^pro^ bound state (annihilation). Right panel: corresponding COM–COM distribution functions as obtained in the HREM simulations of the bound state. The green circles refer to the initial pose as obtained from Autodock4 software.^19^

In Figure 4, we show the (convolution) work distributions *P*(*W*|*F*) and the COM–COM distance distribution functions for all 21 ligands. These two probability distributions, obtained from the NE and HREM simulations, respectively, are the two ingredients used in delivering the predicted standard dissociation free energy of the ligand–3CL^pro^ complex. The combined work distributions serve for the calculation of the Jarzynski or EM functional, while the COM–COM histograms are used for the evaluation of the correction due to the binding site volume.^55^ The latter is estimated as *V*_site_ = (4/3)π (2σ)^3^, where σ^2^ is the variance of the HREM-determined COM–COM histogram, yielding the standard state correction Δ*G*_vol_ = *RT* ln(*V*_site_/*V*_0_) with *V*_0_ = 1661 Å. All ligands are electrically neutral at the physiological pH with the exception of **2913**, which has a positive charge on the sp^3^ nitrogen. For the latter ligand, we applied the finite-size correction to the annihilation free energy for both the bound and unbound states as described in refs (54, 55).

The convolution work distributions (Figure 4, left) exhibit a marked non-Gaussian character (see also AD tests reported in Table 2), leaving hence only eqs 3 or 10 for the free energy estimate. For Δ*G*_EM_, in all cases, we have used *c* = 3. The COM–COM ligand–3CL^pro^ distance distributions (Figure 4, right) denote, in general, a rather large binding site volume, with distance oscillations extending for nearly 4 Å in several cases (e.g., **pari**, **27**, **op3**, **19**, **79**), indicating either a shallow and wide binding site pocket in 3CL^pro^ and/or a significant ligand conformational activity of the ligand in the binding site. As we shall see later on, these peculiar features of 3CL^pro^ binding make the dissociation free energy prediction inherently less accurate. It is worth noting that, in some cases, the docking-determined COM–COM distance, corresponding to the initial pose, is significantly different from the most-likely HREM-determined COM–COM distance, corresponding to the peak of the distribution.

**Table 2:** Standard Dissociation Free Energy Estimates (in kcal/mol) for the 21 Ligands Shown in Figure 2^b^

ligand	Δ*G*_J_^0^	Δ*G*_EM_^0^	Δ*G*_vol_	AD_conv_	AD_u_	AD_b_	Δ*G*_q_	Δ*G*_exp._
pari	10.7 ± 0.6	8.1 ± 4.1	–2.4	490.8	0.40	0.37	–8.6 ± 0.9	n/a
opa	9.4 ± 0.5	6.5 ± 3.1	–2.5	315.0	0.18	1.14	0.3 ± 0.8	n/a
ml	9.0 ± 0.7	9.0 ± 1.9	–3.2	131.1	0.15	0.45	–0.4 ± 0.6	7.9^a^
op3	8.8 ± 0.8	6.9 ± 1.8	–2.2	79.5	0.11	0.48	–2.5 ± 0.8	n/a
27	8.3 ± 0.5	8.1 ± 0.9	–2.7	165.6	0.40	0.54	–1.3 ± 0.3	n/a
39	7.6 ± 1.5	6.2 ± 2.8	–2.5	559.5	0.40	1.39	–1.8 ± 0.4	n/a
mma	7.4 ± 0.8	6.8 ± 1.7	–3.4	55.6	0.37	0.36	0.2 ± 0.4	n/a
ppa	7.3 ± 0.8	4.7 ± 2.5	–3.7	278.2	0.16	0.99	–3.6 ± 2.4	n/a
op2	7.1 ± 2.0	6.3 ± 3.1	–2.6	776.2	0.39	2.03	0.2 ± 0.6	n/a
dolu	6.7 ± 1.6	5.0 ± 3.4	–3.9	190.7	0.74	0.51	0.3 ± 2.0	n/a
19	6.5 ± 0.7	5.2 ± 1.5	–1.9	138.5	0.26	0.54	–3.2 ± 0.6	n/a
30	6.1 ± 0.9	5.1 ± 1.4	–3.5	116.7	0.38	0.43	–1.1 ± 0.3	n/a
mp2	5.8 ± 0.6	3.2 ± 1.4	–3.4	40.7	0.82	0.36	1.8 ± 1.2	n/a
mpa	4.3 ± 1.2	3.6 ± 2.9	–3.7	113.4	0.77	0.49	–1.5 ± 0.4	n/a
77	4.0 ± 0.4	2.3 ± 1.7	–3.0	141.7	0.17	0.53	–0.8 ± 2.4	n/a
n27	3.9 ± 0.6	2.9 ± 1.3	–2.5	114.0	0.42	0.46	–1.1 ± 0.4	n/a
79	3.7 ± 0.9	2.4 ± 0.9	–2.8	142.6	1.91	0.54	–0.4 ± 0.8	n/a
nml	3.1 ± 0.4	1.5 ± 0.5	–3.1	52.5	0.68	0.58	1.0 ± 0.9	n/a
1d45	5.4 ± 0.8	4.6 ± 1.1	–2.9	223.8	0.65	0.22	1.0 ± 0.9	10.0
0b12	9.3 ± 0.9	7.0 ± 1.6	–3.1	183.8	0.50	0.31	1.0 ± 0.9	7.46
2913	5.8 ± 0.8	5.5 ± 1.6	–2.8	201.5	0.63	0.52	1.0 ± 0.9	7.0

a The experimental value refers to the SARS-CoV 3CL^pro^ inhibition.^15^

b Δ*G*_J_, Δ_EM_, Δ*G*_vol_, AD_conv_, AD_u_, AD_b_, and Δ*G*_q_ refer to the Jarzynski free energy estimate; the EM-based free energy estimate; the volume correction; the AD normality test for *P*(*W*|*F*), *P*_u_(−*W*|*G*), and *P*_b_(*W*|*A*); and the electrostatic contribution to the dissociation free energy.

**Figure 5:**
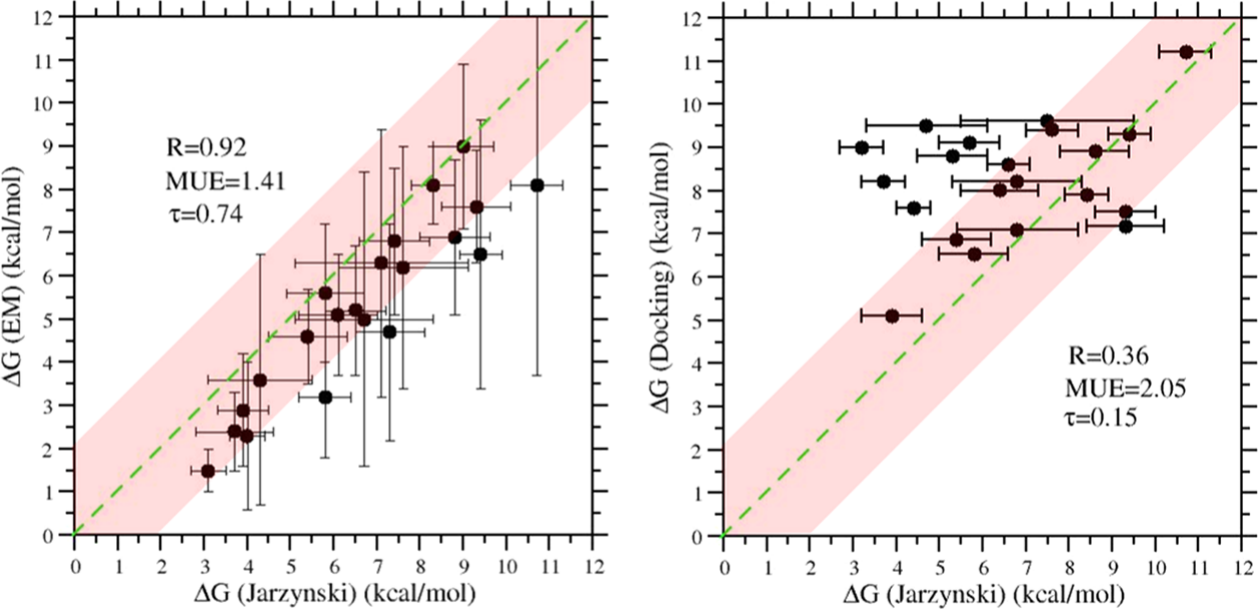
Correlation diagram for the Jarzynski- and EM-based dissociation free energy estimates (left) and for the Jarzynski- and Autodock-based dissociation free energies of the 3CL^pro^ ligands in Figure 2.

In Table 2, we report the results for the computed standard dissociation using the vDSSB approach. Ligands are sorted from the most powerful (**pari**, Jarzynski estimate), of the predicted low nanomolar affinity (20 nM), to the weakest (**nml**), of the millimolar activity (5 mM), as resulting from Δ*G*_J_. The Δ*G*_J_ and Δ*G*_EM_ estimates appear, in general, strongly correlated (see Figure 5), although the latter shows a systematic downshift from a few fractions of kcal/mol to more than 2 kcal/mol for **pari** and **op3**. In general, the Jarzynski estimate is more precise but less accurate than Δ*G*_EM_ based on Gaussian mixtures.^55^ For dissipative NE processes, the Jarzynski estimate very likely remains biased^75^ despite the statistics boosting on the left tails of the work distribution obtained by combining the independent RVs corresponding to the growth and annihilation work of the ligand.

Δ*G*_EM_, on the other hand, while in principle more accurate and unbiased,^50^ is significantly less precise. We recall that the error in the free energy estimates is obtained by bootstrapping independently the growth and annihilation work sample (each containing *n* = 540 work values) and then combining the bootstrapped data to form the convolution *P*(*W*|*F*). For Δ*G*_EM_, the error goes as [1/(*n*/*c*)]^1/2^, i.e., it increases with the number of components (*c* = 3 in our case). EM appears to be sensitive to bootstrapping fluctuations in the *P*_u_(−*W*|*G*) and *P*_b_(*W*|*A*) distributions, producing a rather large variance for the calculation of Δ*G*_EM_ by eq 10. In general, the Jarzynski estimate is found, with no exception, within the confidence interval of the EM-based estimate. One could hence propose as consensus value for the estimate and for the confidence level of the arithmetic mean of the two estimates and errors. A comparison with the experimental results is possible for compound ML (ML188). The measured standard dissociation free energy for the ML188-SARS-CoV 3CL^pro^ complex was found to be^15^ 7.9 kcal/mol, which competes favorably with the consensus vDSSB value of 9.0 kcal/mol found for the strictly related ML188-SARS-CoV-2 complex. For the ligands with known activity, i.e., **1d45**, **0b12**, and **2913**, the consensus value agrees satisfactorily with the experimental counterpart for **0b12** (Δ*G*_c_ = 8.1 kcal/mol, Δ*G*_exp._ = 7.46 kcal/mol) and **2913** (Δ*G*_c_ = 5.7 kcal/mol, Δ*G*_exp._ = 7.0 kcal/mol), while it differs significantly for **1d45** (Δ*G*_c_ = 5.0 kcal/mol, Δ*G*_exp._ = 10.0 kcal/mol). However, while **1d45** is labeled as a noncovalent binder of SARS-CoV-2 3CL^pro^ according to the Covid-19 moonshot activity data,^17^ the same compound was found to be a potent covalent inhibitor with approximately the same dissociation free energy (Δ*G*_exp._ = 10.3 kcal/mol) for the highly homologous SARS-CoV 3CL^pro^.^12^ Covalent binding (that is not accounted for in vDSSB of FEP-based techniques) may explain the observed difference between the experimental and calculated dissociation free energies for **1d45**-3CL^pro^ interaction.

In Figure 5, we report the correlation plots of the Jarzynski estimates with the EM and Autodock4 estimates. Jarzynski–EM correlation is strong, as measured by the Pearson coefficient *R* and the Kendall rank coefficient τ. The mean unsigned difference (MUE) is 1.4 kcal/mol, corresponding to a systematic underestimation of Δ*G*_EM_ with respect to Δ*G*_J_. Free energy estimates obtained with Autodock4 exhibit a rather unexpected significant correlation with vDSSB estimates.

The predicted dissociation free energy range for the 21 ligands goes from 11 to 5 kcal/mol with Autodock4 and from 11 to 3 kcal/mol for the vDSSB Jarzynski estimate, with a surprising agreement for **pari** (highest docking affinity) and **79** (lowest docking affinity) compounds. It should be noted that, except for **pari** and **79**, Autodock predicts dissociation free energy in a range of less than 3 kcal/mol for all other ligands. Probably, the narrow spread in the Autodock4 prediction is due to the smoothing induced by the use of implicit solvent along with the default Gasteiger–Marsili charges^78^ on polar atoms. Absolute values of Gasteiger–Marsili charges on such atoms are in fact significantly smaller than those of the AM1/BCC charges and the AMBER99SB charges used in vDSSB for the ligand and the protein, respectively. Nonetheless, given the low computational cost of Docking, Autodock4 results are remarkable indeed, both in the pose prediction and estimation of the dissociation free energy.

### 5.2 Binding Features in 3CL^pro^

As discussed in Section 4.4, the alchemical protocol prescribes the turning off and on in the sequence of the electrostatic and Lennard–Jones ligand–environment interactions, so that these two contributions to the dissociation free energy can be single out. In Table 2, we report the electrostatic contribution to the dissociation free energy, computed as the sum of the discharging free energy of the ligand in the bound state and the recharging free energy of the ligand in the bulk. The estimates have been done in all cases using eq 4 on the individual electrostatic work samples. As can be seen, such contributions are, in general, small and often negative, with electrostatic interactions being indifferent to or opposing the binding. As far as electrostatics is concerned, for many ligands, the bulk water is hence a more favorable environment than the protein-binding site.^20,32,55^ Since all predicted dissociation free energies are positive, the binding contribution must come from the sum of the ligand’s annihilation and growth Lennard–Jones contributions. The latter is the main chemical–physical determinant for the cavity work and hydrophobic interactions, in general. The fact that hydrophobic interactions are very often those driving the ligand–protein association is due to the heterogeneous nature of the receptors’ binding sites, systematically exposing a mixture of hydrophilic and hydrophobic residues or moieties. 3CL^pro^ makes no exception to this rule.

In Table 3, we report in detail the binding features of the five most potent and four weakest ligands, as assessed by the contact probability between the ligand and the protein residues of domain I + II of 3CL^pro^ obtained from the HREM simulations of the bound state. A ligand is assumed to be in contact with a given protein residue if any ligand–residue atom–atom distance is found below 4.5 Å. Values of 1 for the contact probabilities in Table 3 imply that the ligand has been found in contact with the given residues in all HREM-sampled configurations during the 25 ns simulation of the target state.

**Table 3:**
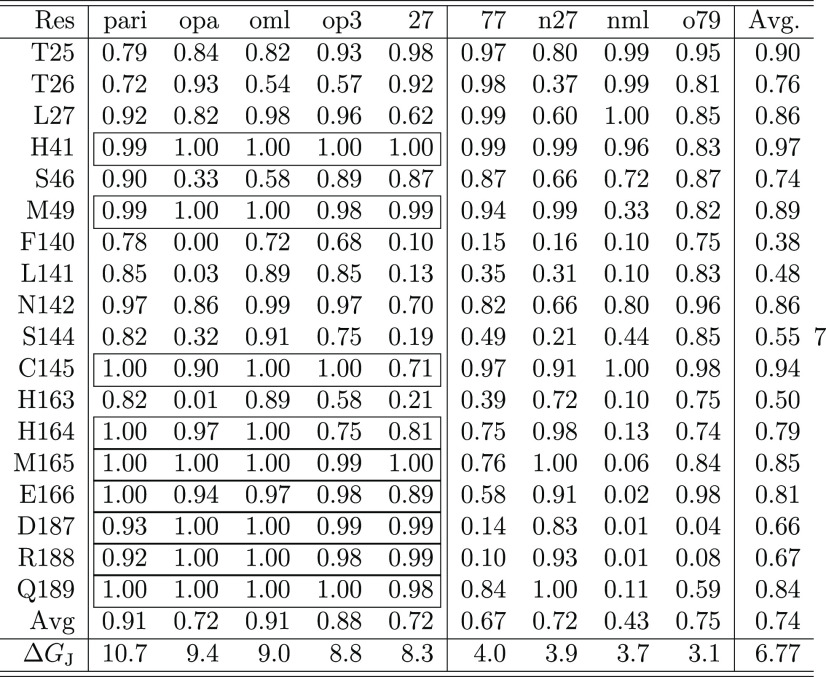
Residue Contact Probability (See the Text) in 3CL^pro^ for the Some Representative Ligands Reported in Figure 2

The high number of vicinal residues with significant average contact probabilities (>0.5) and their mixed character (about half of them can be classified as hydrophobic) is again an indication of the wideness and low specificity of the 3CL^pro^ proteolyitic site. Not surprisingly, years of medicinal chemistry research, after the SARS-CoV 2003 outbreak, were not sufficient for identifying nanomolar or sub-nanomolar 3CL^pro^ noncovalent inhibitors, designing mostly Michael inhibitors with an electrophilic warhead.^4,10,12^ As previously discussed, the nanomolar ligand 1d45^17^ very likely is a mild noncovalent ligand for the SARS-CoV-2 main protease and its strength is due to a postreaction involving a covalent bond on the cysteinate, as found for the SARS-CoV highly homologous 3Clpro.^12^

Based on the reported data, we can attempt to propose a common binding pattern in 3CL^pro^ that might be of help in designing better noncovalent inhibitors for this important viral target. All tested ligands appear to interact strongly with the catalytic dyad H41–C145, with stronger interaction found, in general, for the most potent binders. Persistent hydrophobic interactions in the potent ligands (left part of the table) are those referring to residues L27, M49, and H164 with the histidine residues systematically involved in stacking interactions with the ligand planar moieties. Weak binders (right part of the table) show significantly smaller contact probabilities for these nonpolar or weakly polar residues. Remarkable differences between strong and weak binders are also seen in correspondence to the polar residues E166, D187, R188, and Q189, for which all of the five best binders have approximately unitary contact probability. Very likely, these exposed residues, located on the segment immediately preceding the loop connecting the two subunits in the 3CL^pro^ monomer (see Figure 1a), help to reduce or annihilate the penalty from the electrostatic contribution to the dissociation free energy. These data, in combination with the free energy data of Table 2, are suggestive for an amphiphilic pharmacophore design that is capable of interacting favorably with the polar residues 187–189, with the catalytic dyad, and with M49, L27, and M165.

**Figure 6:**
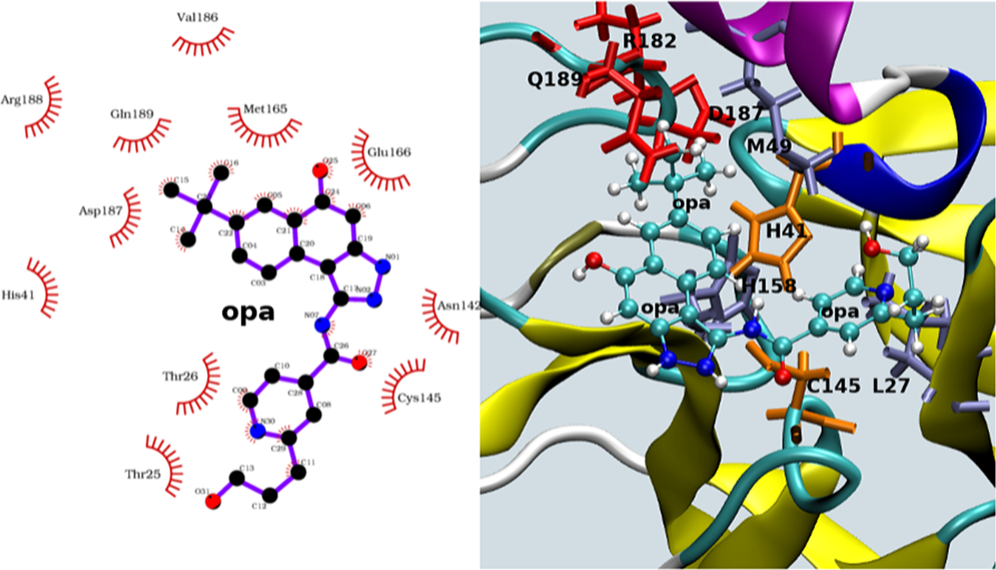
Left: 2D representation^79^ of the binding site of the **opa**–3CL^pro^ complex. Right: corresponding 3D representation.^80^ Hydrophobic and polar residues are in blue and red, respectively. The catalytic dyad, H41–C145, is in orange.

In Figure 6, we report as an example of the two-dimensional (2D) (generated using Ligplot^79^) and 3D (generated using VMD^80^) NPT equilibrated structure of the binding site of the **opa**–3CL^pro^ complex.

## 6 Concluding Remarks

In this contribution, we have described vDSSB, a new nonequilibrium alchemical technique that exploits enhanced sampling and work distribution convolution to effectively emulate the double-system single-box approach with increased efficiency, accuracy, and precision. vDSSB, as described in the present study, can be implemented with no need for code modification in the most popular MD programs supporting NE alchemical simulations (e.g., GROMACS^81^ or AMBER^82^).

The collected information provides valuable clues and indications for 3Cl^pro^ binding and, possibly, inhibition, as they are based on extensive and advanced molecular dynamics simulations on HPC facilities involving several tens of microseconds of simulations in total using state-of-the-art atomistic force fields and explicit solvents. Nonetheless, when dealing with compounds with pharmacological interest for the ongoing Covid-19 pandemic, caution is a must and some caveats regarding our results are in order.

First, in ref (9), we have shown that the protonation state of catalytic dyad has a very limited impact (fraction of kcal/mol) on the predicted binding free energies (using Autodock4) for about 100 tested ligand–3Cl^pro^ complexes. In this study, all calculations have been hence done assuming both C145 and H41 in their neutral state. Although in explicit solvent atomistic simulations, the electrostatic screening at short distance is much more effective with respect to that resulting from an implicit solvent approach, the effect of protonation state on binding affinity modulation cannot be ruled out.

Second, a weak point of all alchemical theories, whether equilibrium (such as FEP or TI) or nonequilibrium (vDSSB), is the computation of the standard state correction related to the binding site volume. In FEP, this correction is estimated from the difference between the free energy of imposing the restraint potential (usually a harmonic function involving translational, orientational, and conformational degrees of freedom of the ligand) in the binding site at full ligand coupling and the free energy of releasing that restraint at zero coupling. In the strong restraint limit, this difference can be shown^43,83^ to be equal to *RT* log(*V*_site_/*V*_0_). While the zero-coupling contribution is computed analytically, the bound-state free energy cost of the restraint in virtually all FEP applications for absolute binding free energy determination is inappropriately computed again via FEP using a stratification where the restraints are progressively switched on, in a few windows and in a few nanoseconds in total at best, with the ligand lingering in the presumed binding site with the presumed conformation/orientation. In NEW-vDSSB, only a COM–COM constant restraint potential is imposed along the alchemical coordinate, with, hence, no biasing on whatsoever the ligand orientational/conformation that is sampled (in the fully coupled initial states) using powerful enhanced sampling approaches. In this case, the binding volume correction is likely to produce fewer artifacts (related to, e.g., a wrong ligand pose) with respect to FEP in *de novo* absolute binding free energy predictions. Nonetheless, Δ*G*_vol_ is based on an approximated calculation of the elusive binding site volume and standard dissociation free energy could be hence significantly affected.

## Supporting Information Available

Compressed archive containing (i) table with the SMILES codes of the 21 ligands, (ii) PrimaDORAC-generated tpg/pmr files (orac format), (iii) NPT equilibrated structure of the TIP3P-solvated bound state for all ligand–receptor pairs (in pdb format), and (iv) best-docked complexes obtained using Autodock4; and HREM trajectories (target state, in PDB format not including water) for all ligand–receptor complexes listed in Table 3 that can be downloaded from the link indicated in ref (84) (ZIP)
